# Different Approaches to Address Bullying in KiVa Schools: Adherence to Guidelines, Strategies Implemented, and Outcomes Obtained

**DOI:** 10.1007/s11121-020-01178-4

**Published:** 2020-10-24

**Authors:** Eerika Johander, Tiina Turunen, Claire F. Garandeau, Christina Salmivalli

**Affiliations:** 1 INVEST Research Flagship, Department of Psychology and Speech-Language Pathology, University of Turku, Turku, Finland; 2 Shandong Normal University, Jinan, China

**Keywords:** KiVa antibullying program, Indicated actions, Bullying, Intervention, Long term, Implementation fidelity, Outcome, Confronting, Non-confronting, Follow-up

## Abstract

We examined the extent to which school personnel implementing the KiVa® antibullying program in Finland during 2009–2015 systematically employed the program-recommended approaches (*confronting* or *non-confronting*), used one or the other depending on the bullying case (*case-specific* approach), or used their *own adaptation* when talking to perpetrators of bullying, and whether they organized follow-up meetings after such discussions. In addition to investigating adherence to program guidelines, we tested how effective these different approaches were in stopping bullying. Finally, we tested the contribution of follow-up meetings and the number of years KiVa had been implemented in a school to the effectiveness of the interventions, using reports from both school personnel and victimized students. The data were collected annually across 6 years via online questionnaires and included responses from 1221 primary and secondary schools. The school personnel were more likely to use the confronting approach than the non-confronting approach. Over time, rather than sticking to the two program-recommended approaches, they made adaptations (e.g., combining the two; using their own approach). Two-level regression analyses indicated that the discussions were equally effective, according to both personnel and victimized students, when the *confronting*, *non-confronting*, or a *case-specific* approach had been used. The discussions were less effective when the personnel used their *own adaptation* or *could not specify* the method used. Perceived effectiveness was higher in primary school and when follow-up meetings were organized systematically after each intervention, but unrelated to the number of years KiVa had been implemented.

Over the past decades, growing awareness of the negative outcomes of school bullying (Reijntjes et al. 2010) has in many countries led to normative regulation, such as schools being required to have a policy, or an action plan against bullying (Salmivalli 2018). School personnel are thus faced with a demand to do *something* to address bullying. At the same time, numerous antibullying programs have been developed and evaluated in different parts of the world (Gaffney et al. 2019). Such programs often combine preventive actions (such as student lessons or improved supervision) with targeted interventions (i.e., procedures for intervening in actual bullying cases, such as discussions with the students involved). Evaluation studies have, however, mainly estimated the effects of whole programs (without distinguishing prevention from intervention components), and the few studies that have compared the effectiveness of different approaches in targeted interventions only assessed short-term effectiveness on the basis of a single student informant (Garandeau et al. 2014, 2016). Consequently, we know little about the relative effectiveness of different approaches used when a case of bullying has already occurred, and even less about how school personnel implement guidelines provided to address such cases. The present study investigates the extent to which school personnel implementing the KiVa® antibullying program (Kärnä et al. 2011a) in Finland employ the program-recommended approaches (confronting vs. non-confronting) when discussing with bullying perpetrators, how this changes over a period of 6 years, and how effective the chosen approaches (whether program-recommended or something else) are perceived to be by the school personnel and by the students who have been victimized.

## Implementing the KiVa Antibullying Program

KiVa is an evidence-based antibullying program which, after a randomized controlled trial in 2007–2009 (Kärnä et al. 2011b, 2013), became available for all schools providing basic education (grades 1–9) in Finland. In a few years, more than 90% of schools in the country had adopted the program. KiVa includes universal, preventive actions directed at all students (such as student lessons) and indicated actions targeted at students directly involved in bullying cases. The present study focuses on the latter.

Each school implementing the program has a KiVa team, consisting of three or more adults working in the school (often teachers), who intervene when a case of bullying comes to the attention of school personnel. A series of discussions is organized, including separate meetings with the victimized child and the perpetrator(s) of bullying. The KiVa teacher manuals provide detailed guidelines for two alternative approaches that can be employed when discussing with the perpetrator(s): so-called confronting and non-confronting approaches (Garandeau et al. 2014). It is strongly recommended in the guidelines that follow-up discussions are organized about 2 weeks after the first meeting, to make sure that the bullying has stopped. It is also emphasized that follow-up discussions should be scheduled already in the first meetings with the students involved, so that they understand that the situation will be monitored and there will be check-up in the near future.

During the randomized controlled trial of KiVa, the indicated actions taken led to a positive outcome (bullying decreasing or stopping completely) in as many as 98% of cases (Garandeau et al. 2014). However, conducting strictly controlled evaluation trials is one thing, bringing interventions to scale is another. Evidence-based methods are not necessarily implemented at all; they may be adapted or only partially employed by users (Moore et al. 2013; Sainio et al. 2018; Stirman et al. 2013). The reasons for adaptation may include lack of time, limited resources, lack of information, lack of appropriate training, or strong beliefs regarding the (non-)effectiveness of a particular strategy (Durlak and DuPre 2008; Haataja et al. 2015; Moore et al. 2013; Ringwalt et al. 2003). A growing body of research suggests that the closer the implementation of an intervention follows its original design, the better the obtained outcomes (Durlak and DuPre 2008; Wilson and Lipsey 2007); the debate on whether guidelines should be carefully followed or whether adaptations might lead to better outcomes is, however, ongoing (Cross and West 2011; Parekh et al. 2019).

## Intervening in Cases of Bullying: Which Approach Is Most Effective?

Antibullying policies or programs may guide school personnel to “intervene immediately when detecting bullying” or “have serious discussions with the students involved”—but the specific procedures that they should follow often remain unclear. This is concerning, since, according to students, teacher interventions often fail in putting an end to bullying (Davis and Nixon 2011; Fekkes et al. 2005; for a review, see Rigby 2014). These studies did not specify the approaches employed; however, they suggest that knowledge on effective ways to intervene in bullying is urgently needed.

Intervention strategies that have been investigated in the literature include two major approaches: a direct, confronting approach and an indirect, non-confronting approach (Garandeau et al. 2014). In the confronting approach, the emphasis is on setting clear limits for unacceptable behavior by telling the perpetrators that their behavior has come to the attention of the school personnel, is not tolerated, and must stop immediately (see Olweus 1993). The non-confronting approach was derived from methods such as the Method of Shared Concern (Pikas 1989) and the Support Group Method (Robinson and Maines, 2008). In that approach, the adult aims to increase bullies’ empathy for their victim by sharing their own concern for the victimized peer’s situation, without blaming the perpetrator(s) or even mentioning that they are the source of harm. The objective is to get them to share the adult’s concern and to offer solutions to improve the situation.

Although school personnel tend to prefer using confronting, authoritarian approaches over non-confronting ones (Bauman et al. 2008; Burger et al. 2015; Power-Elliott and Harris 2012), evidence of their superior effectiveness is lacking. Indeed, most studies only examined the effect of a single approach (van der Ploeg et al. 2016; Young 1998) or utilized very small samples and no testing of statistical differences between the strategies (Thompson and Smith 2011). In their meta-analysis, Ttofi and Farrington (2011) found that disciplinary (i.e., confronting) strategies were typical of effective programs. As pointed out by the authors, however, this finding was to some extent consequential to the large effects of the Olweus Bullying Prevention Program (OBPP) as a whole. The program includes actions taken at the school and classroom levels, as well as at the level of individual students involved in bullying. At the individual level, OBPP recommends that school personnel adopt a disciplinary/confronting strategy by having “serious discussions with bullies.” The analysis by Ttofi and Farrington compared the effects of whole programs involving vs. not involving specific components (such as disciplinary strategies with bullies); the extent to which disciplinary strategies or some other components included in the OBPP were responsible for the large effects obtained is, however, not known. Also, non-confronting work with the bullies was absent in Ttofi and Farrington’s coding of the program components.

Only two studies directly compared the two approaches, both in the context of the randomized controlled trial (RCT) of the KiVa antibullying program in Finland (Garandeau et al. 2014, 2016). In the RCT, half of the intervention schools were instructed and trained to use the confronting approach, whereas the other half were instructed and trained to use the non-confronting approach. The effectiveness of the approaches was first evaluated by asking the victims in a follow-up meeting, about 2 weeks after the intervention, whether the bullying had stopped (Garandeau et al. 2014). According to the victims, bullying had stopped in 78.2% of the cases. Neither approach was shown to be overall more effective than the other, after controlling for the level of schooling (primary versus secondary school), type of aggression (e.g., verbal, physical, relational, online), and the duration of victimization (for how long it had been going on). However, some factors were found to moderate the relative effectiveness of the two approaches. Whereas the two approaches were equally successful in cases of long-term victimization and in primary schools, the confronting approach was slightly more successful than the non-confronting approach in cases of short-term victimization and in secondary schools. The latter finding is in contrast to suggestions presented in the literature (Pikas 1989; Rigby and Griffiths 2010) that the non-confronting approach might be especially suited to be used with adolescents.

Bullying perpetrators’ perceptions of the interventions and the consequences of these perceptions for their intention to change their behavior were examined in another study (Garandeau et al. 2016). The bullies’ intention to change their behavior was highest when they felt that the teacher had *both* condemned the bullying behavior and tried to arouse their empathy for the victim, rather than employing only one of the two strategies.

Based on existing research, there is no evidence that one approach is more effective than the other. However, because previous studies have only measured the short-term effectiveness of the approaches using single student informants (Garandeau et al. 2014, 2016), more knowledge is needed regarding the long-lasting effects of the approaches. Also, without controlling what the school personnel actually did in the discussions, it is uncertain whether they implemented the approaches exactly as instructed. In addition, using bullies’ self-reported intention to change their behavior as a measure for the effectiveness (Garandeau et al. 2016) may be problematic as social desirability bias may influence the reports.

## The Present Study

Even when research-based guidelines regarding how to address bullying are available, it is not self-evident that they are followed. Teachers may adapt the recommended approaches in ways they think better meet their needs, or they may employ completely different approaches. In the present study, we address the adherence vs. adaptation issue (Cross and West 2011; Parekh et al. 2019) for the very first time in the area of bullying interventions. Using data from Finnish schools followed over a period of 6 years, our first aim is to investigate the extent to which their personnel systematically employed the program-recommended approaches (*confronting* or *non-confronting*), used one or the other depending on the bullying case (*case-specific* approach), or used their *own adaptation* when talking to perpetrators of bullying, and whether they organized follow-up meetings after such discussions—as recommended in the program.

In addition to investigating implementation adherence over time, we examine the perceived effectiveness of the different approaches. Only two studies to this date have directly compared the confronting and non-confronting approaches that are recommended in KiVa manuals. One study measured only short-term effectiveness—about 2 weeks after the intervention using victim reports (Garandeau et al. 2014); the other study considered bullies’ intention to change but did not assess actual or perceived changes in behavior (Garandeau et al. 2016). We examine the perceived effectiveness of different strategies chosen, using reports from both school personnel and students who had been victimized. Importantly, our comparison includes, in addition to the two approaches, schools’ own adaptations, and we investigate the contribution of organizing follow-up meetings. Finally, we take into account the level of schooling (primary vs. secondary) and the number of years the school has been implementing the KiVa program.

## Method

### Sample and Procedure

**Fig. 1:**
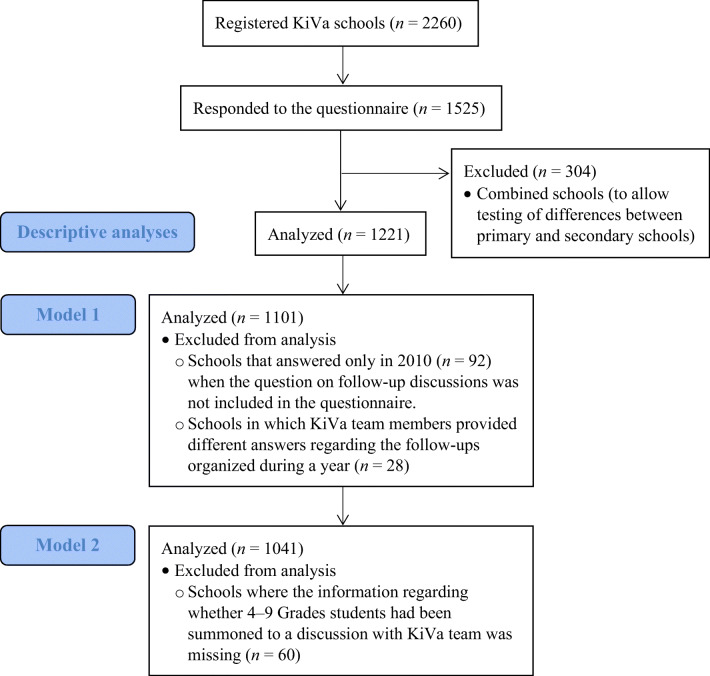
Flow diagram of the schools included in the analysis

Data for the present study came from Finnish schools that were implementing the KiVa antibullying program between 2009 and 2015 and responded at least once to the annual online questionnaire (about program implementation) starting in 2010 (Fig. 1). Out of the 2260 registered KiVa schools, the KiVa teams from 1525 schools (68%) responded at least once to the questionnaire. Combined schools (304) were excluded from the analyses to allow testing of differences between primary and secondary schools, leaving us with data from 1221 schools from all around Finland: 978 of them were primary (grades 1–6) and 243 secondary (grades 7–9) schools. Primary schools were underrepresented, as there were 2197 (85%) primary and 388 (15%) secondary schools in Finland during the years 2009–2015 (Official Statistics of Finland 2019), as compared with the 80% and 20% in the present sample.

At the end of each school year, registered KiVa schools are invited to respond to three online questionnaires. One questionnaire is for the teachers delivering KiVa student lessons in the school, one is for the school’s KiVa team (one or several members of the team can respond), and one is for the students. The present study uses data from the two latter questionnaires. In the schools where more than one KiVa team member responded, personnel-perceived effectiveness of the approach used was averaged across their responses.

Data from the 1221 schools were used for descriptive analyses. As the question regarding school personnel’s organization of follow-up discussions was only included in the online questionnaire since 2011, data from 1101 schools were used for the multilevel model, in which personnel-perceived effectiveness of the discussions is the outcome variable (model 1, see below).

Students responded to the surveys during regular school hours, using school-specific passwords to log in. The question, on whether the bullying experienced by the student had been addressed by adults at school, was asked only from the students in grades 4–9.

Among the 1101 schools, students from 1041 schools in grades 4–9 reported being summoned to a discussion with the KiVa team because they had been bullied (*n* = 38,931, that is 9.4% of the total sample of 416,323 respondents in grades 4–9 in those schools). Consequently, only data from those schools were used for the multilevel analysis in which student-perceived effectiveness of the discussions is the outcome variable (model 2, see below). Among the 1221 schools used in descriptive analyses, 466 schools (i.e., schools’ KiVa teams) responded once, 299 schools responded twice, 199 schools responded three times, 142 schools responded four times, 81 schools responded five times, and 34 schools responded six times to the questionnaire. Among the 1101 schools used in model 1, 438 schools responded once, 284 schools responded twice, 177 schools responded three times, 129 responded four times, and 73 schools responded five times to the questionnaire. Among the 1041 schools used in model 2, 434 schools (both KiVa teams and students) responded once, 253 schools responded twice, 173 schools responded three times, 115 schools responded four times, and 66 schools responded five times to the questionnaire.

### Measures

#### Level of Schooling

The information about the level of schooling (0 = *primary*, 1 = *secondary*) is provided by the schools when they start implementing the KiVa program.

#### Number of Years of KiVa Implementation

The number of years schools had been implementing the KiVa program was calculated as the difference between the year they had originally registered as program users and each measurement year (the year in which the response was provided). The range of responses was 1–6.

#### Approaches Used

The KiVa teams were asked to indicate which approach they had used in handling the cases of bullying during the past school year. The response option were as follows: (1) *consistently the confronting approach*, (2) *consistently the non-confronting approach,* (3) *either the confronting or the non-confronting approach depending on the bullying case*, (4) *either the confronting or the non-confronting approach depending on the team member*, (5) *the school’s own adaptation that was neither the confronting nor the non-confronting approach*, and (6) *I do not know*. The last option is herein referred to as “an unspecified method”. Prior to the analyses, responses three and four were compounded into one category, labeled “*case-specific approach*,” and five dummy-coded variables (0 = *school did not use the method*, 1 = *school used the method*), were created to represent these five response categories, namely confronting approach, non-confronting approach, case-specific approach, own adaptation, and unspecified method.

#### Organizing Follow-up Discussions

The KiVa teams were asked to indicate how often, if at all, they organized follow-up discussions. The response options to the question “Has your school’s KiVa team arranged follow-up discussions to make sure that the bullying has stopped?” were the following: (1) *no*, (2) *occasionally*, or (3) *in all cases.* Three dummy-coded variables were created to represent these three categories in the analyses.

#### Personnel-Perceived Effectiveness of the Discussions

The KiVa teams were asked to evaluate the effectiveness of their interventions during the past school year. Responses to the question “In your opinion, to what extent have the discussions led to a desired outcome (that is, ceasing of the bullying)?” were given on a 5-point scale (0 = *not at all or very poorly*, 1 = *rather poorly*, 2 = *I do not know*, 3 = *rather well*, 4 = *very well*).

#### Student-Perceived Effectiveness of the Discussions

Students, who reported that the bullying they had experienced (as victims) was addressed by the adults at school, were asked whether the intervention had an effect on their situation. Response options to the question “When you had been bullied, did the adult intervention affect your situation?” were the following: (1) *the situation did not change at all, I was still bullied*, (2) *since then I was bullied less or the bullying stopped completely*, and (3) *since then I was bullied more.* For the analyses, responses one and three were compounded into one category “*did not change at all/increased*” and a dummy-coded variable (0 = *did not change at all/increased*, 1 = *decreased/stopped*) was created. Individual student responses within each school were averaged to create a school-level mean variable of student-perceived effectiveness.

### Analysis Plan

To take into account the nested structure of the data (time points, or response years, nested within schools—there were several responses from each school given in different years), a two-level regression analysis with random intercepts was used to test the effects of four predictors, namely the number of years the school had implemented KiVa, the approach used, organizing follow-up discussions, and the level of schooling, on the personnel- and student-perceived effectiveness of the discussions. Analyses were conducted using *M*plus 8 (Muthén and Muthén 1998) and the robust version of maximum likelihood estimation (MLR). Response years were the within-level units, while schools were the between-level units. Within-level predictors included the number of years the school had implemented KiVa, the approach used, and organizing follow-up discussions; the level of schooling was a between-level predictor. With respect to the dummy variables, the confronting approach, organizing the follow-up discussion in all cases, and primary schools were used as reference categories. Because the question on follow-up discussions was not included in the questionnaire until 2011, answers to this question were missing from 2010. When the KiVa team members provided different answers regarding the follow-ups organized during a year, these responses (*n* = 28) were excluded from the two-level regression analyses.

## Results

### Descriptive Statistics

**Fig. 2:**
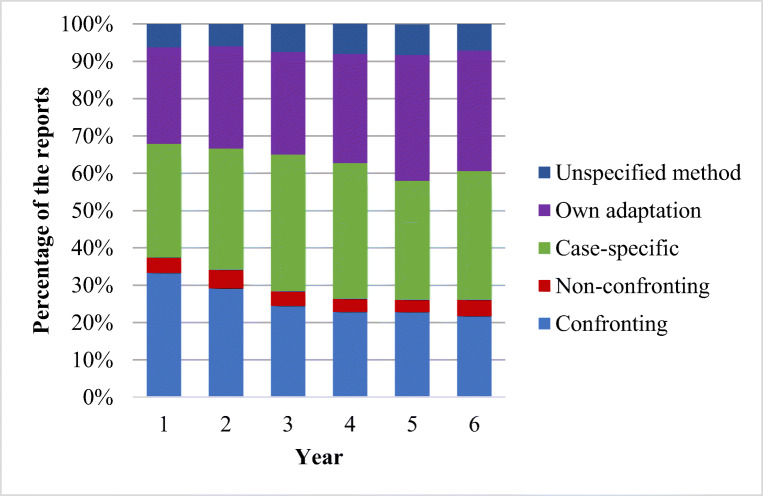
Used approaches during the different years of program implementation. Case-specific = using C or NC depending on bullying case

The proportion of schools using each of the five approaches varied across years (Fig. 2). Among the 1221 schools, 21.7 to 33.4% reported using the confronting approach, 30.5 to 36.7% reported using the case-specific approach, and 25.9 to 33.7% reported using their own adaptation. In contrast, only 3.3 to 4.9% of the schools reported using the non-confronting approach, and in 5.9–8.2% of the schools, the KiVa team members could not specify which approach they had used.

Cross-tabulation revealed a significant relationship between the number of years the schools had been implementing KiVa and the approach used, *χ*^2^ (20) = 37.40, *p* = .01. During the first year of the program implementation, the use of the confronting approach was more common than would have been expected by chance (33.4% of the schools, adjusted standardized residual = 4.4), whereas the use of the case-specific approach was less common than would be expected by chance (30.5% of the schools, adjusted standardized residual = − 2.0). However, during the fourth year of the program implementation, the use of the confronting approach occurred less than expected by chance (22.9% of the schools, adjusted standardized residual = − 2.2), and by the fifth year, the use of the school’s own adaptation was common (33.7% of the schools, adjusted standardized residual = 2.3). There is thus a trend from following the recommended strategies in the early years of program implementation towards more adaptation later on.

The within-school correlations and descriptive statistics for the study variables are presented in Table 1. The use of the confronting approach was positively correlated, and the use of the school’s own adaptation was negatively correlated with personnel-perceived effectiveness, whereas the use of unspecified method was negatively correlated with both personnel- and student-perceived effectiveness of the intervention discussions. Organizing follow-up meetings systematically after every discussion was positively correlated and organizing them only occasionally was negatively correlated with both personnel- and student-perceived effectiveness, whereas not organizing follow-up discussions at all was negatively correlated only with the personnel-perceived effectiveness. Student-perceived effectiveness tended to be higher in primary school than in secondary school. The longer the schools had been implementing KiVa, the less likely they were to use the confronting approach and the more likely they were to use their own adaptation. However, organizing the follow-up discussions was more likely in schools with more years of KiVa implementation.

**Table 1:** Within-school correlations and descriptive statistics of study variable

Variable	1	2	3	4	5	6	7	8	9	10	11	12	13
1. Student-perceived effectiveness	−												
2. Personnel-perceived effectiveness	.08	−											
3. Primary schools	.26***	.04	−										
4. Secondary schools	−.26***	−.04	−1.00	−									
5. Years in KiVa	.04	.01	.01	−.01	−								
6. Confronting	.02	.10***	−.09***	.09***	−.10***	−							
7. Non-confronting	.04	.03	.03	−.03	−.01	−.13***	−						
8. Case-specific	.01	.02	−.01	.01	.02	−.43***	−.15***	−					
9. Own adaptation	−.03	−.05*	.07**	−.07***	.07**	−.38***	−.13***	−.45***	−				
10. Unspecified method	−.04	−.14***	.03	−.03	.02	−.16***	−.06**	−.19***	−.17***	−			
11. No follow-up	.00	−.21***	.06**	−.06**	−.08***	−.07**	−.04	−.11***	.03	.31***	−		
12. Follow-up held occasionally	−.09***	−.14***	−.12***	.12***	.01	−.09***	−.04	−.01	.12***	−.01	−.09***	−	
13. Follow-up held in all cases	.08***	.23***	.09***	−.09***	.03	.12***	.05*	.06**	−.12***	−.14***	−.39***	−.88***	−
M	.74	3.17	.79	.21	3.22	.27	.04	.34	.28	.07	.04	.16	.80
SD	.19	.58	.40	.40	1.52	.44	.20	.47	.45	.25	.19	.37	.40
ICC	.17	.12											

Note: *n* = 2418; correlation coefficients between binary variables are phi coefficients

*ICC* intraclass correlation

***p < .001

**p < .01

*p < .05

The mean of personnel-perceived effectiveness of the discussions was 3.17 (scale 0–4), and the mean of student-perceived effectiveness was 0.74 (0 = the situation did not change, or bullying had increased, 1 = bullying had decreased, or stopped). This means that overall, the discussions were quite effective according to both school personnel and students who had been victimized. The intraclass correlations for personnel- and student-perceived effectiveness of the interventions (ICC = .17 and .12, respectively) indicated that 12–17% of the total variance in perceived effectiveness was due to differences between schools. That is, some schools were overall doing better than others with respect to successfully intervening in bullying. However, most of the variation in perceived effectiveness was due to differences within schools across years.

### Predictors of Effectiveness of the Discussions

#### Personnel-Perceived Effectiveness

In the first two-level model, we predicted the personnel-perceived effectiveness of the discussions (model 1, see Table 2). With respect to the between-level part of the model, the school personnel in secondary schools perceived the discussions to be significantly less effective than the personnel in primary schools (*b* = − 0.060, *p* = 0.04). However, the level of schooling explained only 1.0% of the between-level variance in personnel-perceived effectiveness.

**Table 2:** Multilevel regression analyses for predicting the perceived effectiveness of the discussions

	Model 1: KiVa teams		Model 2: victimized students	
Variable	*b*	SE	*b*	SE
Secondary schools	− .060*	.029	− .123***	.009
Years in KiVa	− .004	.007	.005	.003
Non-confronting	− .008	.055	.018	.020
Case-specific	− .059	.032	− .008	.009
Own adaptation	− .090**	.033	− .025*	.011
Unspecified method	− .251***	.063	− .050*	.023
No follow-up	− .590***	.090	.007	.030
Follow-up held occasionally	− .218***	.030	− .023*	.011
*R*^2^ Within	.096***	.016	.011*	.005
*R*^2^ Between	.010	.010	.527***	.124

*Note*: *n* for model 1 = 2418 (within), 1101 (between). *n* for model 2 = 2249 (within), 1041 (between). Reference categories are the confronting approach, organizing the follow-up discussion in all cases and primary schools

***p < .001

**p < .01

*p < .05

The within-level predictors explained 9.6% of the within-school variance in personnel-perceived effectiveness. The number of years the school had implemented KiVa was not significantly related to personnel-perceived effectiveness. Regarding the approach used, the effects of the non-confronting and the case-specific approach on personnel-perceived effectiveness did not significantly differ from the effects of the confronting approach. However, when the schools had used their own adaptation (*b* = − .090, *p* = .006), or when they could not specify the method used (*b* = − .251, *p* < .001) the personnel perceived the discussions to be significantly less effective than when the schools had used the confronting approach. When the schools did not organize follow-up discussions (*b* = −.590, *p* < .001), or organized them only occasionally (*b* = − .218, *p* < .001), the personnel perceived the discussions to be significantly less effective than when follow-up discussions were organized systematically (i.e., in all cases).

#### Student-Perceived Effectiveness

Model 2 included the same predictors as model 1 and student-perceived effectiveness of the discussions as the outcome variable (Table 2). Consistent with the results of model 1, the students in secondary schools perceived the discussions to be significantly less effective than the students in primary schools (*b* = − .123, *p* < .001). The level of schooling explained as much as 52.7% of the between-level variance in student-perceived effectiveness.

The within-level predictors explained only 1.1% of the within-school variance in student-perceived effectiveness. Consistent with the results of model 1, the number of years schools had implemented KiVa was not significantly related to student-perceived effectiveness. Also, student-perceived effectiveness did not differ from the confronting approach when the non-confronting or case-specific approach had been used, but the use of the school’s own adaptation (*b* = −.025, *p* = .018) or unspecified method (*b* = −.050, *p* = .029) was perceived to be less effective. Furthermore, the effectiveness was lower when the schools had organized follow-up discussions only occasionally (*b* = −.023, *p* = .041) compared to a systematic organization of follow-up discussions.

#### Comparisons Between Primary and Secondary Schools

Finally, to test whether the effects of the within-level predictors on the personnel-perceived and on the student-perceived effectiveness of the discussions were equal in primary and secondary schools, multigroup comparisons were conducted. A chi-square difference test with the Satorra-Bentler correction was used to compare the constrained model—where the relationships between each predictor variable and the personnel- and student-perceived effectiveness of the discussions were constrained to be equal in primary and secondary schools—to a freely estimated model—where the relationships were allowed to differ. The models fit equally well for personnel-perceived (Δ*χ*2 (7) = 13,314 *p* = .07) and student-perceived effectiveness (Δ*χ*2 (7) = 5247 *p* = .64), indicating that the effects of within-school predictors did not differ significantly between primary and secondary schools.

## Discussion

Existing literature on the school personnel’s use of different approaches to address bullying is limited, and little is known about the relative effectiveness of different approaches. Using data from Finnish schools implementing the KiVa antibullying program, followed for a period of 6 years, this study examined whether the school personnel conducting targeted interventions (i.e., discussions with bullying perpetrators) used the program-recommended approaches vs. their own adaptations, and whether they organized follow-up meetings after such discussions. The present study shed light on the question of implementation fidelity, and more specifically on the adherence vs. adaptation issue (Parekh et al. 2019), by investigating how effective these different approaches were in reducing bullying, using reports from both school personnel and students. In addition, we examined how the effectiveness of the actions taken was influenced by the level of schooling, the duration of KiVa implementation, and the schools’ organizing of follow-up meetings. Whether the effects differed in primary schools vs. secondary schools was also investigated.

In line with previous studies showing that most teachers reported they would respond to bullying cases with authority-based interventions (e.g., Bauman et al., 2008; Burger et al., 2015), the school personnel implementing the KiVa program strongly favored the use of the confronting over the non-confronting approach. Among the participating schools, 21.7 to 33.4% (across 6 years) used only the confronting approach, 3.3 to 4.9% used only the non-confronting approach, and 30.5 to 36.7% used a case-specific approach (i.e., either of the two depending on the situation). The rest of the schools used their own adaptation, or they could not specify the method they had used.

Overall, the most common way to handle cases of bullying was to follow the program guidelines by using either a confronting or a non-confronting approach (58–68% of the schools). However, the school personnel’s preference evolved over the years. During the first year of program implementation, their most common choice was the confronting approach, but using the school’s own adaptation became more frequent over the years. In the fifth year of program implementation, the most common choice was the school’s own adaptation. This shift might be related to the timing of the training for the program. The longer the schools had been implementing the program, the more time had passed since the pre-implementation training. Although implementation manuals were available to them, the personnel may lack the time to consult them, or may even have forgotten that they contained information on the recommended targeted interventions.

According to both student and personnel reports, the discussions were overall quite effective in reducing bullying, and their effectiveness did not vary depending on whether the school personnel had consistently used the confronting or the non-confronting approach, or either one depending on the situation. This finding is in line with previous studies, providing no evidence that either the confronting or the non-confronting approach was overall more effective than the other (Garandeau et al. 2014, 2016). However, the discussions were less effective when the school personnel had used their *own adaptation* or when they *could not specify* which approach they had used. Thus, increasing adaptation did not add to the efficacy of the intervention, quite the contrary. This is in line with studies suggesting that higher adherence to the program’s original design results in better outcomes (Durlak and DuPre 2008).

Our data lacks information regarding the nature of the adaptations and the reasons for them. Adaptations are often made due to lack of time or limited resources (e.g., Moore et al. 2013) and sometimes they could be necessary in a given organization. In the case of targeted bullying interventions, however, it is more likely that adaptations were made in response to challenging cases; when faced with such cases, the personnel may have felt that doing something slightly different from the recommended approaches was necessary or preferable. Alternatively, they may have chosen to do the targeted discussions “their own way” because it felt easier or more comfortable to them, or because they believed that it would be more efficient. Even when adaptations are appropriate, the school personnel may lack the skills to apply them in a way that would increase the effectiveness of the intervention; therefore, if making adaptations is necessary for the schools, possibility for guidance on how to make them effectively might help the schools reach the desired outcomes.

The discussions were least effective when the school personnel could not specify which method they had used. They may have continuously switched between different strategies or they may have simply tried to do something, without a clear idea of what should be done, which could explain the low effectiveness.

Rather than being associated with the use of the confronting or non-confronting approach, the effectiveness of the interventions was found to depend strongly on the systematic organization of follow-ups. The school personnel perceived the discussions as less effective when they did not organize follow-up discussions at all, or when they organized the follow-up discussion only occasionally, compared to when they organized follow-up discussions in all cases. A clear practical implication can be drawn: For the discussions to be effective, organizing follow-up meetings to ensure that the bullying has stopped is essential. When follow-ups are organized, it conveys the message to the bullying children (from the first meeting) that the adults take bullying seriously. The awareness that their behavior will be monitored, which is less likely with one-time discussions, is probably necessary for perpetrators to stop their bullying behavior. The anticipation of the follow-up meeting is likely to affect the bullying students’ behavior and explain why organizing follow-ups is associated with better outcomes. It is indeed good news that school personnel increased their use of follow-up meetings over time, even if they otherwise adhered less to the recommended approaches.

Overall, the discussions were perceived to be less effective in secondary schools compared to primary schools. This is not in line with the findings of Garandeau et al. (2014) who investigated short-term effectiveness and found no indication that the discussions would be less effective in secondary schools than in primary schools. However, in that study, the duration of victimization (how long the bullying had been going on) was included as a predictor along with the level of schooling, and it was negatively associated with the success of the interventions. Thus, the effect of the level of schooling may have been confounded with duration of victimization. In secondary schools, it may be more likely that the bullying has lasted longer and become chronic, which might make it more difficult to intervene. Also, the effects of the whole KiVa program on bullying and victimization have been smaller and less consistent in secondary schools than in primary schools (Kärnä et al. 2013). Interventions might be less effective in secondary schools due to developmental changes in the prioritizing of peer reputation. As being popular becomes a priority for many students towards the end of primary school and in secondary school (Dawes and Xie 2014, 2017; LaFontana and Cillessen 2010), and bullying might be effective in reaching that goal (de Bruyn et al. 2010; Duffy et al. 2017), bullies may be less motivated to restrain their behavior in secondary school.

The number of years schools had implemented the KiVa program did not have an impact on the effectiveness of the discussions. This is surprising; over time, the KiVa teams should gain experience in addressing bullying cases and become more comfortable in implementing the interventions, which in turn should be reflected in heightened effectiveness. However, it is unknown whether the team members have remained the same or changed over time.

### Limitations

Approximately one-third (33%) of the KiVa teams did not respond at all to the questionnaire during the 6 years of the study. Therefore, data from 738 registered KiVa schools were lacking. We do not know whether these schools implemented the targeted intervention discussions and, if so, which approach they used.

The data analyzed in this study came from schools that were free to choose between different approaches rather than instructed to use one or the other. It might be that certain types of schools were more likely to choose some specific approach, and that the effectiveness of the chosen approach depended more on the features of those schools than on the approach itself. For instance, the schools that are most committed to antibullying work might be most likely to follow the guidelines and use the recommended approaches. However, many schools varied the approach they used across the study years, and therefore, this should not have affected the results to a large extent. Also, only 12–17% of the variation in perceived effectiveness was due to differences between schools.

The data used in this study lacked information about what was done when the schools used their own adaptation and why they used them. In future studies, investigating the type of adaptations schools made to the recommended approaches and the reasons for these adaptations would increase our understanding of the link between fidelity of implementation and effectiveness of targeted interventions.

The present study focused on the perceived effectiveness—by personnel and by students—of the used approaches. It did not consider strictly objective measures of whether the bullying had stopped. Finally, the approaches recommended in the program were limited to a confronting and a non-confronting one—other strategies, such as the restorative approach (Morrison 2002), or direct sanctions (Thompson and Smith 2011) were thus not considered in this study. A thorough investigation of the relative effectiveness of different antibullying strategies would include comparisons among a wider variety of methods.

## Conclusions

The approach that school personnel spontaneously choose when intervening in bullying is not necessarily the most effective one. The school personnel perceived the discussions to be equally effective when they had used a confronting, a non-confronting, or a case-specific approach. However, most of them chose to use the confronting, rather than the non-confronting approach. Thus, even though both of the recommended approaches are perceived to be equally effective, one of them was almost never chosen. Future research should seek to identify the reasons why most school staff appears to avoid using non-confronting approaches.

The longer the schools had been implementing the program, the more the personnel started to use their own adaptations. This happened despite the fact that schools which employed their own adaptation were less satisfied with the outcome of their interventions. Whereas increasing adaptation of the recommended approaches did not add to their efficacy, organizing follow-up meetings systematically after each intervention had a positive effect. Our findings emphasize the importance of adherence to evidence-based methods rather than the superiority of a specific approach as such, for the effectiveness of targeted antibullying interventions.
