# Correction to: Different Approaches to Address Bullying in KiVa Schools: Adherence to Guidelines, Strategies Implemented, and Outcomes Obtained

**DOI:** 10.1007/s11121-020-01201-8

**Published:** 2021-01-14

**Authors:** Eerika Johander, Tiina Turunen, Claire F. Garandeau, Christina Salmivalli

**Affiliations:** 1 INVEST Research Flagship, Department of Psychology and Speech-Language Pathology, University of Turku, Turku, Finland; 2 Shandong Normal University, Jinan, China

**Correction to: Prevention Science**

10.1007/s11121-020-01178-4

The original version of this article unfortunately contained a mistake in Table 1. The values under ICC row were invertently changed to “.17 and .58”, instead of “.17 and .12”.

The original article has been corrected.

